# Quantitative muscle ultrasound is useful for evaluating secondary axonal degeneration in chronic inflammatory demyelinating polyneuropathy

**DOI:** 10.1002/brb3.812

**Published:** 2017-09-15

**Authors:** Keiichi Hokkoku, Kiyoshi Matsukura, Yudai Uchida, Midori Kuwabara, Yuichi Furukawa, Hiroshi Tsukamoto, Yuki Hatanaka, Masahiro Sonoo

**Affiliations:** ^1^ Department of Neurology Teikyo University School of Medicine Itabashi‐ku Tokyo Japan

**Keywords:** chronic inflammatory demyelinating polyneuropathy, demyelination, denervation, muscle ultrasound, secondary axonal degeneration

## Abstract

**Introduction:**

In chronic inflammatory demyelinating polyneuropathy (CIDP), exclusion of secondary axonal degeneration is challenging with conventional methods such as nerve conduction study (NCS), needle electromyography, and nerve biopsy. Increased echo intensity (EI) and decreased muscle thickness (MT) identified on muscle ultrasound (MUS) examination represent muscle denervation due to axonal degeneration in neurogenic disorders, suggesting MUS as a new tool to detect secondary axonal degeneration in patients with CIDP.

**Methods:**

EI and MT of abductor pollicis brevis, abductor digiti minimi, and first dorsal interosseous muscles were measured in 16 CIDP patients. Raw values were converted into *z*‐scores using data from 60 normal controls (NCs).

**Results:**

Six of 45 muscles showed abnormally high EI and low MT, suggesting denervation following secondary axonal degeneration. These six muscles belonged to two patients with long disease history, unresponsiveness to treatment, and long interval from onset to initial therapy. There were no significant differences in EI and MT (*p* = .23 and .67, respectively) between the CIDP and NC groups, although NCS results revealed obvious demyelinating abnormalities in all CIDP patients, suggesting the fact that muscle structures will be preserved, and EI and MT will not change unless secondary axonal degeneration occurs in CIDP.

**Conclusion:**

MUS is a promising tool for evaluating secondary axonal degeneration in patients with CIDP.

## 1 INTRODUCTION

Chronic inflammatory demyelinating polyneuropathy (CIDP) is an immune‐mediated, acquired demyelinating neuropathy that progresses over more than 2 months in association with symmetrical weakness, impaired sensation, and absent or diminished tendon reflexes following a progressive or relapsing–remitting course (Bouchard et al.*,* 1999 *;* Harbo, Andersen, Jakobsen, 2008; Said, 2006). Although the main pathology in CIDP is segmental demyelination, several studies demonstrated coexistence of axonal degeneration following segmental demyelination termed secondary axonal degeneration (Bouchard et al.*,* 1999; Boukhris et al.*,* 2004 *;* Said, 2006; Sghirlanzoni et al.*,* 2000). Differentiating segmental demyelination from secondary axonal degeneration in patients with CIDP is essential as the latter suggests unresponsiveness to medication and unfavorable prognosis (Bouchard et al.*,* 1999, 1999; Iijima et al.*,* 2005 *;* Said, 2006; Sghirlanzoni et al.*,* 2000). However, ruling out axonal degeneration from segmental demyelination is challenging with conventional methods (Harbo et al.*,* 2008; Tankisi, Pugdahl, Johnsen, & Fuglsang‐Frederiksen, 2007 *;* Tankisi et al.*,* 2005). In nerve conduction studies (NCSs), not only axonal degeneration but also conduction block or increased temporal dispersion due to segmental demyelination result in decreased compound muscle action potential (CMAP) amplitude (Harbo et al.*,* 2008; Kuwabara, Ogawara, Misawa, Mori, & Hattori, 2002 *;* Tankisi et al.*,* 2005 *,* 2007). Needle electromyography (EMG) can reveal axonal degeneration by detecting abnormal spontaneous activities such as fibrillation potentials or positive sharp waves due to denervation in patients with CIDP (Sghirlanzoni et al.*,* 2000 *;* Tankisi et al.*,* 2007). However, the procedure is invasive and is still debated on its usefulness in CIDP (Harbo et al.*,* 2008; Rubin, 2012; Tankisi et al.*,* 2007). While nerve biopsy appears the most accurate approach to detect axonal degeneration, its use is also limited by its invasiveness and limitation to sensory nerves in most cases (Harbo et al.*,* 2008).

Muscle ultrasound (MUS) has become a useful screening tool for neuromuscular disorders as a noninvasive, easily accessible, and relatively inexpensive method (Gunreben & Bogdahn, 1991 *;* Heckmatt, Leeman, & Dubowitz, 1982 *;* Pillen et al.*,* 2007; Walker, Cartwright, Wiesler, & Caress, 2004). Neurogenic disorders cause structural changes to muscle tissues due to denervation following axonal degeneration. After denervation, muscle tissues are replaced with fat and fibrous tissues, which can be detected as increased echo intensity (EI) and decreased muscle thickness (MT) on MUS (Gunreben & Bogdahn, 1991; Pillen, Arts, & Zwarts, 2008 *;* Pillen et al.*,* 2007; Simon et al.*,* 2015; Walker et al.*,* 2004). Hence, MUS may be utilized as an alternative tool to elucidate secondary axonal degeneration by assessing denervation. To the best of our knowledge, there are no reports investigating MUS findings in patients with CIDP, with a specific focus on denervation. In this study, we quantitatively investigated the utility of MUS for evaluating denervation in patients with CIDP.

## 2 MATERIALS AND METHODS

### 2.1 Subjects

Patients with CIDP who were admitted to our department from December 2014 to November 2016 were prospectively enrolled. All patients with CIDP fulfilled the criteria for definite CIDP determined by the European Federation of Neurological Societies/Peripheral Nerve Society (EFNS/PNS) (Joint Task Force of the EFNS and the PNS, 2010). Patients with a history of other neuromuscular disorders, orthopedic diseases, or metabolic diseases such as diabetes that can affect muscles and nerves were excluded. Normal controls (NCs) comprising 60 healthy volunteers (30 males and 30 females; age, 51.9 ± 19.8 years; range, 20–90 years) were also recruited to construct normal values for MUS. All patients and healthy volunteers provided written informed consent for the procedures. The ethics committee of the Teikyo University School of Medicine approved this study (approval number: 15‐052, October 2015).

### 2.2 Study protocol

The Medical Research Council (MRC) grading scale was used to measure the muscle strength of abductor pollicis brevis (APB), abductor digiti minimi (ADM), and first dorsal interosseous (FDI) in all patients by an experienced neurologist (MS). The NCSs on median and ulnar nerves and the MUS on APB, ADM, and FDI were performed on the clinically more affected side determined by the same neurologist (MS). Both NCSs and MUS were conducted on the same day or with an interval of less than 1 week. All MUS procedures were performed by the same examiner (KH), whereas NCSs were performed by several trained doctors or technicians other than KH. The MUS and NCS results were withheld from the examiner of the other test. In addition, detailed medical history including disease duration, severity of weakness, and medications was also blinded, although the diagnosis of the patient was known to every examiner. In NCs, MUS was conducted only on the dominant hand.

### 2.3 Nerve conduction studies

Motor NCSs were performed on median and ulnar nerves in all subjects via standard methods of our laboratory using an MEB‐2200 EMG device (Nihon Kohden, Tokyo, Japan). The CMAPs of the APB were recorded in median NCS with stimulations at the wrist and elbow. The CMAPs of the ADM and FDI were recorded simultaneously in ulnar NCS with stimulations at the wrist, below and above the elbow, the upper arm, and the Erb's point. Peak‐to‐peak CMAP amplitude, distal latency, distal CMAP duration, and nerve conduction velocity were evaluated. Abnormal findings were determined using normal data obtained at our laboratory. In the ulnar NCS with FDI recordings, only CMAP amplitudes were evaluated as normal values for other parameters were not available. Skin temperature was measured on the palm and was maintained at 33°C or higher throughout the examination.

### 2.4 MUS measurements

**Figure 1:**
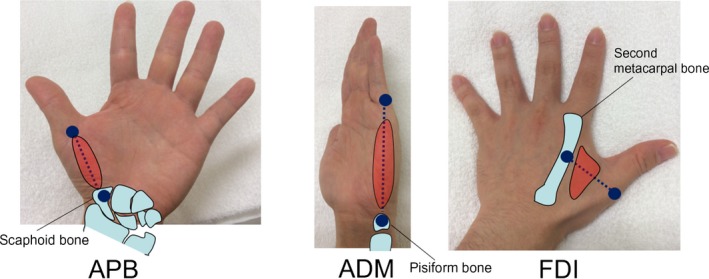
MUS recording sites for APB, ADM, and FDI muscles. The probe was placed along the line connecting the midpoint of the volar aspect of the first metacarpophalangeal joint and the volar prominence of the scaphoid bone for APB, along the line connecting the midpoint of the fifth metacarpophalangeal joint and pisiform bone for ADM, and on the dorsal surface of the hand perpendicular to the axis of the second metacarpal bone at the level of the first metacarpophalangeal joint for FDI. MUS, muscle ultrasound; APB, abductor pollicis brevis; OP, opponens pollicis; ADM, abductor digiti minimi; FDI, first dorsal interosseous

**Figure 2:**
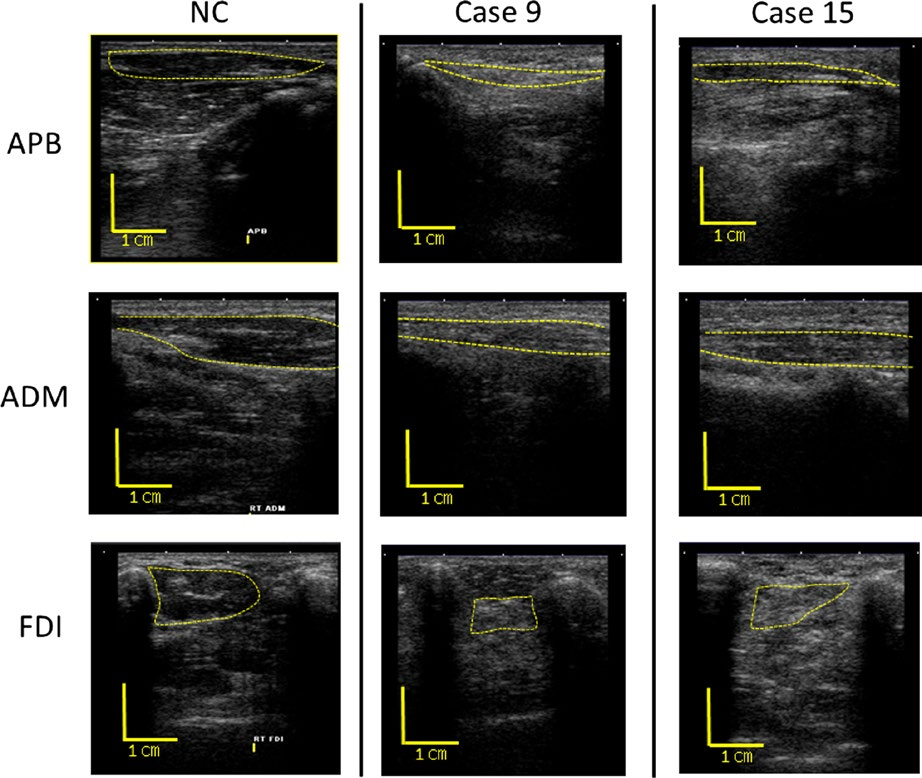
Visual MUS findings. NC: Typical views and findings of each muscle in an NC. Each muscle was surrounded by a broken line. The parenchyma of every muscle appeared black, indicating low echo intensity. Cases 9 and 15: The muscle parenchyma of each muscle appeared white (indicating high echo intensity), and the size of muscles was smaller than that in an NC. MUS, muscle ultrasound; APB, abductor pollicis brevis; FDI, first dorsal interosseous; NC, normal control

MUS was performed in APB, ADM, and FDI. All MUS examinations were conducted using a Nemio MX ultrasound device and a 7.5–11 MHz linear array probe with a peak frequency at 11 MHz (Toshiba Corporation, Tokyo, Japan). Equipment settings were maintained as follows during the entire examination: gain, 84 dB; compression, 55; time‐gain compensation in neutral position; and three focal points at 1.5, 2.0, and 2.5 cm. All subjects laid in the supine position. The probe was placed along the line connecting the midpoint of the volar aspect of the first metacarpophalangeal joint and the volar prominence of the scaphoid bone for APB, along the line connecting the midpoint of the fifth metacarpophalangeal joint and the pisiform bone for ADM, and on the dorsal surface of the hand perpendicular to the axis of the second metacarpal bone at the level of the first metacarpophalangeal joint for FDI (Figure 1). Orientation of the probe was determined to ensure adequate attachment to the recording site and avoid excessive compression, which could induce fluctuations in EI and MT. Typical views and normal findings of each muscle are shown in Figure 2. Muscle parenchyma at all sites, which appeared black, denoted low echo intensity. Images were stored as joint photographic experts group files with a resolution of 640 × 480 pixels. We did not evaluate dynamic MUS findings such as fasciculations in this study.

### 2.5 Quantitative assessment of EI and MT

For the quantitative assessment of EI, raw images were transferred to a personal computer and measured with computer‐assisted gray‐scale analysis using the standard histogram function in Adobe Photoshop Elements 13 (Adobe Systems, San Jose, CA). Region of interest (ROI) was set to cover the entire muscle cross‐section in the ultrasound screen, and the mean gray‐scale value of the ROI was measured and expressed as a value between 0 (black) and 255 (white). The measurement was repeated three times and the mean value was adopted. MT was measured using an electronic caliper equipped with an ultrasound device. The thickest location of the muscle belly was measured in each muscle. All quantitative assessments were performed by the same examiner (KH).

### 2.6 Statistical analysis

To evaluate the test–retest reliability of consecutive quantitative MUS assessments performed by the same examiner (KH), intraclass correlation coefficient (ICC) (1, 1) for EI and MT was assessed in 10 randomly selected subjects in the NC group. The same examiner (KH) performed three separate tests for each subject. The first and second tests were performed on the same day with an interval of at least 3 hours, and the third test was performed 1–3 days after the last test. The ICC (1, 1) was calculated from the three examinations. Stepwise multiple linear regression analysis was performed in the NC group to evaluate whether gender and age were independent predictors of EI and MT, as previously reported (Pillen et al., 2008). Based on the analysis, NCs were classified into several groups according to gender as well as age (40 years or less, 41–60 years, and 61 years or more). Raw EI and MT values in the NC and CIDP groups were converted into *z*‐scores for standardization using values from the corresponding NC group. Normal ranges for EI and MT values were determined according to the *z*‐scores of the NC group. The *z*‐scores of both groups were tested using the Mann–Whitney *U* test to determine significant differences between the CIDP and NC groups.

## 3 RESULTS

### 3.1 Characteristics of the study subjects

In total, 16 patients with CIDP (8 males and 8 females; age, 59.3 ± 17.2 years; range, 19–76 years) were enrolled. Disease duration was 27.5 ± 33.2 months (range, 3–120 months). The MRC scales of APB, ADM, and FDI were 3.3 ± 1.1, 3.0 ± 1.0, and 3.2 ± 1.0, respectively. Eight patients were newly diagnosed with CIDP. Of the eight patients with recurrent CIDP, seven patients had previously received intravenous immunoglobulin therapy (IVIg). However, the last course had been administered more than 5 months prior to entry into this study. Three patients were treated with corticosteroids and/or immunosuppressants as maintenance therapy. One patient had not received any medications previously (Table 1).

**Table 1:** Summary of clinical data of patients with chronic inflammatory demyelinating polyneuropathy

	CIDP (*n* = 16)
Male/Female	8/8
Age (y/o) (mean ± *SD*)	59.3 ± 17.2
Range	19–76
Disease duration (month)	
Mean ± *SD*	27.5 ± 33.2
Range	3–120
Median	14
MRC scale (mean ± *SD*)	
APB	3.3 ± 1.1
ADM	3.0 ± 1.0
FDI	3.2 ± 1.0
Initial case	8
Recurrent case	8
Treatment in recurrent cases	
IVIg	4
IVIg + CS + IS	1
IVIg + CS	1
IVIg + IS	1
No medication	1

APB, abductor pollicis brevis; ADM, abductor digiti minimi; CIDP, chronic inflammatory demyelinating polyneuropathy; FDI, first dorsal interosseous; IVIg, intravenous immunoglobulin; CS, corticosteroids; IS, immunosuppressants; MRC, Medical Research Council; *SD*, standard deviation.

### 3.2 NCS

Nerve conduction study results revealed evident demyelinating abnormalities in all study patients. All patients exhibited at least one abnormality in each of the parameters, that is, distal latency, distal CMAP duration, and nerve conduction velocity, thereby fulfilling the electrodiagnostic criteria for definite CIDP diagnosis set by the EFNS/PNS guidelines. Conduction block and increased temporal dispersion, both of which were also found in several patients (1/16, 6/16, and 5/13 patients in the median NCS on APB, ulnar NCS on ADM, and ulnar NCS on FDI, respectively) (Table 2A). Data obtained from the NCS on FDI recording from three patients were excluded from this study due to technical problems.

**Table 2A:** The results of muscle ultrasound assessment in patients with CIDP

Case	Median nerve NCS					Ulnar nerve NCS															
	DL (ms)	Dur (ms)	NCV (m/s) W‐E	CMAP (mV)		DL (ms)	Dur (ms)	NCV (m/s)				CMAP (ADM) (mV)					CMAP (FDI) (mV)				
				W	E			W‐BE	BE‐AE	AE‐UA	UA‐EP	W	BE	AE	UA	EP	W	BE	AE	UA	EP
1	5.1	6.8	26	7.3	6.0	3.8	6.8	45	45	22	25	11.8	10.6	9.8	7.0	5.1^b^	13.3	10.8	10.0	8.6	6.0^b^
2	5.2	6.6	44	10.4	7.8	3.0	6.5	43	33	39	39	15.2	13.1	13.0	10.0	4.6^a^	15.4	7.5^a^	8.2	9.6	4.8^a^
3	6.4	7.8	U	4.8	0^a^	4.4	9.7	36	14	56	57	7.8	6.4^b^	6.0	3.4	2.0	8.3	6.8^b^	6.4	2.8^a^	2.2
4	8.8	8.0	23	5.1	2.3^b^	3.6	7.8	20	22	23	U	10.2	7.1	6.4	2.5	0^a^	6.6	3.8	4.2	2.2	0^a^
5	14.0	8.8	25	6.7	4.4^b^	6.6	10.1	24	16	32	29	6.3	3.7	3.2^b^	3.0	2.3	2.8	2.3	1.3^b^	1.2	1.2
6	6.6	9.0	38	3.4	3.2	3.7	6.4	50	28	51	40	5.7	5.3	5.1	5.1	1.9^a^			U		
7	10.6	13.4	15	4.5	3.5^b^	11.0	14.6	16	8	34	16	5.0	2.1^a^	1.9	1.2	0.70	2.4	1.0^a^	0.90	0.70	0.40
8	6.3	6.6	35	5.0	4.8	4.4	6.8	46	36	40	49	10.5	9.6^b^	9.2	7.3	6.4			U		
9	5.8	6.6	34	1.9	1.3^b^	3.5	6.8	35	23	29	24	6.7	4.2^b^	3.4	3.7	3.1	3.4	2.2^b^	2.0	2.4	2.0
10	6.1	6.8	28	12.0	7.1	3.4	7.2	39	19	29	37	10.0	7.4	5.7	4.5	1.0^a^	16.6	15.8	12.0	8.4	0.90^a^
11	4.7	7.9	37	7.7	7.3	4.8	7.1	44	42	33	28	6.8	6.3	6.2	5.8	3.2^a^			U		
12	4.3	8.0	30	12.8	11.3	4.4	7.3	33	22	29	22	11.6	9.7	8.5	6.7	3.7	11.9	9.2	8.1	8.3	3.8^a^
13	7.9	10.2	49	6.6	5.9	5.9	7.8	42	18	53	66	11.2	10.4	10.2	10.0	9.1	3.8	3.7	3.7	3.6	2.8
14	7.1	7.5	46	12.2	10.9	3.6	5.8	51	24	26	21	18.7	16.7	15.2	14.3	12.1	18.6	15.6	12.1	12.4	11.2
15	4.9	4.8	35	5.8	5.1	3.0	5.4	49	21	28	21	8.8	4.3^b^	4.1	3.4	0.20^a^	8.2	6.6^b^	6.1	5.6	0.20^a^
16	3.6	4.1	40	11.2	6.3	6.1	5.8	55	36	55	56	13.8	7.5	7.2	7.2	6.1	14.4	8.3	8.3	6.8	4.2
Mean	6.7	7.7	31.6	7.3	5.5	4.7	7.6	39.2	25.5	36.2	33.1	10.0	7.8	7.2	5.9	3.8	9.7	7.2	6.4	5.6	3.1
*SD*	2.6	2.1	12.3	3.4	3.1	2.0	2.3	11.3	10.4	11.5	17.4	3.7	3.8	3.6	3.4	3.3	5.7	4.8	3.8	3.7	3.1
	CB: 1/16, increased TD: 4/16					CB: 6/16, increased TD: 6/16 in ADM, CB: 6/13, increased TD: 5/13 in FDI															

Demyelinating abnormalities fulfilling the electrodiagnostic criteria for “definite” according to the European Federation of Neurological Societies/Peripheral Nerve Society (EFNS/PNS) guideline are underlined.

NCS, nerve conduction study; DL, distal latency; Dur, duration; NCV, nerve conduction velocity; CMAP, compound muscle action potential; W, wrist; E, elbow; BE, below elbow; AE, above elbow; UA, upper arm; EP, Erb's point; CB, conduction block; TD, increased temporal dispersion; ADM, abductor digiti minimi; FDI, first dorsal interosseous; U, unavailable.

a Conduction block.

b Increased temporal dispersion.

**Table 2B:** The results of muscle ultrasound assessment in patients with CIDP

Case	MUS (*z*‐score)					
	APB		ADM		FDI	
	EI	MT	EI	MT	EI	MT
1	0.12	−0.62	0.13	−0.23	0.21	0.23
2	−0.44	−1.9	−0.22	0.87	−1.1	−0.08
3	−0.36	−0.31	−0.01	1.3	0.35	0.02
4	0.02	−0.35	−0.72	0.56	−1.1	0.21
5	−0.92	1.3	0.48	0.05	−2.4	−0.03
6	2.3	−0.64	0.18	0.18	U	
7	1.2	0.27	−0.40	−0.95	1.0	0.21
8	1.3	−1.2	0.34	−0.78	U	
9	10.1^a^	−4.3^a^	4.9^a^	−4.8^a^	5.9^a^	−2.7^a^
10	−1.1	0.49	0.42	1.8	2.0	0.47
11	1.4	−0.28	1.3	1.6	U	
12	1.4	−0.64	−0.32	−0.61	0.40	0.96
13	−0.46	−0.28	−0.77	0.28	−0.99	0.56
14	−2.1	−0.39	−0.60	1.8	−2.0	−0.35
15	3.4^a^	−2.1	3.5^a^	−2.9^a^	3.8^a^	−1.5
16	1.6	1.6	0.09	−1.2	−0.02	−029
Mean	1.1	0.20	0.51	−0.18	1.1	−0.20
*SD*	2.8	1.5	1.5	1.7	2.8	1.5
	Mean *z*‐scores of three muscles EI, 0.02 ± 1.1; MT, 0.23 ± 0.82					

MUS, muscle ultrasound; EI, echo intensity; MT, muscle thickness; APB, abductor pollicis brevis; ADM, abductor digiti minimi; FDI, first dorsal interosseous; U, unavailable.

a Abnormally high EI or low MT.

### 3.3 Quantitative assessment of EI and MT

The ICCs (1, 1) of EI and MT displayed substantial agreement among the muscles. The ICCs (1, 1) of EI and MT in the APB, ADM, and FDI were 0.78, 0.74, and 0.80 and 0.85, 0.90, and 0.89, respectively. These results determined that the reproducibility of the quantitative assessment was guaranteed and further experiments were warranted.

In total, 45 (APB, 16; ADM, 16; FDI, 13) and 180 (APB, 60; ADM, 60; FDI, 60) muscles from the CIDP and NC groups, respectively, were included in the final analysis. Stepwise multiple linear regression analysis in the NC group revealed that gender significantly affected the EI of the ADM and FDI as well as the MT of every muscle. Furthermore, within the NC group, age significantly affected the EI of every muscle and the MT of the FDI (Table 3). The raw EI and MT values of the CIDP and NC groups were converted into z‐scores according to the values from the corresponding NC group classified with gender and age (Table 2B).

**Table 3:** Multiple linear regression analysis based on normal controls

Dependent variable	Model			Significant independent variables	Coefficient		
	*R* ^*2*^	*F*	*p value*		β	*t*	*p value*
APB EI	0.20	15.3	<.001	Age	0.46	3.9	<.001
APB MT	0.18	14.1	<.001	Gender	−0.44	−3.8	<.001
ADM EI	0.19	7.8	<.01	Gender	0.34	2.9	<.01
				Age	0.33	2.8	<.01
ADM MT	0.29	25.1	<.001	Gender	−0.55	−5.0	<.001
FDI EI	0.39	38.3	<.001	Age	0.63	6.1	<.001
FDI MT	0.29	13.3	<.001	Gender	−0.51	−4.7	<.001
				Age	−0.27	−2.4	<.05

APB, abductor pollicis brevis; ADM, abductor digiti minimi; FDI, first dorsal interosseous; EI, echo intensity; MT, muscle thickness.

### 3.4 Quantitative MUS in the CIDP group

**Figure 3:**
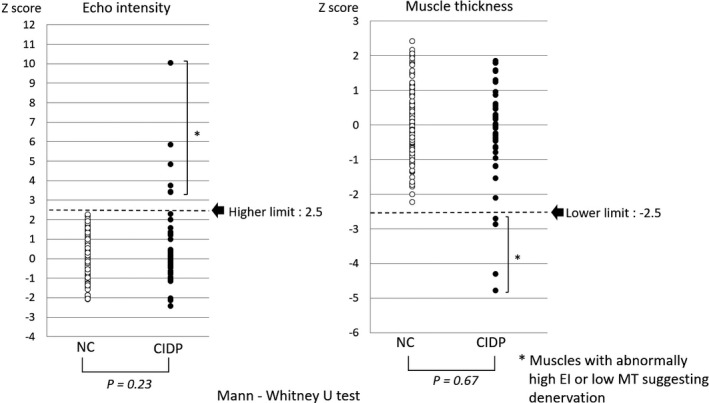
Comparison of EI and MT between the CIDP and normal control groups. Six muscles with abnormally high EI (*z*‐score >+2.5) and four with low MT (*z*‐score <−2.5) were observed, suggesting denervation. No significant difference was observed between the CIDP and NC groups in terms of EI or MT (*p* = .23 and .67 for EI and MT, respectively). EI, echo intensity; MT, muscle thickness; CIDP, chronic inflammatory demyelinating polyneuropathy; NC, normal control

We set the normal range of *z*‐scores as +2.5 for EI and −2.5 for MT, as all values of the NC group fell within this range. In the CIDP group, although most of the EI and MT values fell within this range, there were four muscles with abnormally high EI and abnormally low MT, in addition to two muscles with abnormally high EI. These six muscles belonged to patients 9 and 15 (Table 2B and Figure 3). In both patients, the muscles visually exhibited whiter parenchyma and atrophy compared to the NC group (Figure 2). Patient 9 was a 40‐year‐old woman who had a long disease course with more than 10 years and was unresponsiveness to prednisolone. Her symptoms were gradually progressing, although intravenous immunoglobulin was administered from time to time. Patient 15 was an 82‐year‐old man who was diagnosed with CIDP 3 years before entry to this study. However, he had not been able to receive any medication because of social and economic problems. The number of patients in this study was too small to determine statistical significance; however, the mean disease duration was about four times longer in the two patients compared to the remaining patients with normal MUS findings (88.0 and 18.9 months, respectively). Comparison of the EI and MT *z*‐scores between the CIDP and NC groups revealed that there were no significant differences between the groups for either parameter (*p *=* *.23 and .67 for EI and MT, respectively; Figure 3).

### 3.5 Comparison of NCS and MUS findings

**Figure 4:**
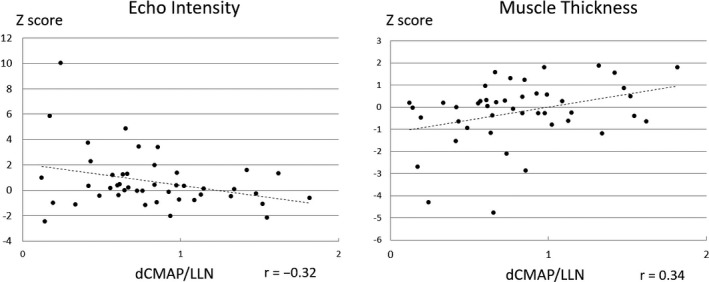
Correlation of dCMAP amplitude with EI and MT. Fair correlations of dCMAP/LLN with both EI and MT was found in the CIDP group. EI, echo intensity; MT, muscle thickness; dCMAP/LLN, the ratio of measured distal compound muscle action potential amplitude to the lower limit value of distal compound muscle action potential amplitude in normal subjects

To standardize the distal CMAP (dCMAP) amplitude values measured in three different muscles, we determined the ratio of the measured values to the lower limit of values obtained from normal subjects (LLN) utilized in our laboratory. There was no significant difference in the dCMAP/LLN ratio between the six muscles with abnormally high EI and/or low MT values and the remaining muscles with normal MUS findings in patients with CIDP (*p *=* *.18 with Mann–Whitney *U* test). There were fair correlations between the dCMAP/LLN ratio and the EI and MT values in patients with CIDP (−0.32 and 0.34, respectively; Figure 4).

## 4 DISCUSSION

### 4.1 Quantitative MUS in patients with CIDP

Several recent studies reported that the determination of EI and MT by quantitative MUS was highly accurate and reproducible in detecting hand muscle denervation (Kim, Seok, & Kim, 2016; Lee et al.,^2016^; Shahrizaila et al., 2017; Simon et al., 2015). MUS findings including EI and MT in denervated hand muscles correlated with the severity of EMG abnormalities and facilitated the discrimination between moderate‐to‐severe muscle denervation and healthy hand muscles (Kim et al., 2016; Pillen et al., 2008; Simon et al., 2015). In this study, the quantitative MUS assessment on small hand muscles in patients with CIDP revealed that six out of 45 muscles with abnormally high EI and/or low MT suggested denervation following secondary axonal degeneration.

In this study, muscle denervation was determined only by quantitative MUS. Needle EMG was not performed in this study with an academic focus due to its invasiveness on small hand muscles. Pillen et al. insisted that MUS was equally or slightly less capable of detecting focal neuropathies than EMG, with a sensitivity estimated at 70% (Pillen et al., 2008). Simon et al. reported that mild denervation changes in EMG were detectable even though EI and MT were within normal ranges (Simon et al., 2015). Hence, our cut‐off values of +2.5 for EI and −2.5 for MT, which excluded all EI and MT values of the NCs, albeit not sensitive, might be specific for muscles with axonal degeneration and suitable for exclusion of muscles without axonal degeneration (Figure 3).

The two patients with abnormally high EI and/or low MT by MUS exhibited several characteristics that could have contributed to the development of secondary axonal degeneration such as the history of a long disease course, unresponsiveness to medication, and the long interval from onset to initiation of therapy (Hahn, Bolton, Zochodne, & Feasby, 1996; Iijima et al., 2005;Kuwabara et al.,^2006^, ^2002^; Said, 2006). The NCSs determined that both patients exhibited overt demyelinating abnormalities that fulfilled the electrodiagnostic criteria for definite CIDP according to the EFNS/PNS guidelines, as shown in other patients with normal MUS findings (Joint Task Force of the EFNS and the PNS, 2010) (Table 2A). These NCS findings indicate that segmental demyelination was responsible for the primary pathophysiology in both patients (Cornblath et al., 1992; Logigian, Kelly, & Adelman, 1994; Van Asseldonk, Van den Berg, Kalmijn, Wokke, & Franssen, 2005), although their MUS findings showed high EI and low MT values, suggesting the existence of axonal degeneration, and that the primary segmental demyelination progressed to secondary axonal degeneration.

### 4.2 Comparison of MUS findings between the CIDP and NC groups

Although the NCS findings of all CIDP patients in this study showed definite demyelinating abnormalities, no significant differences in EI and MT were noted between the CIDP and NC groups. This finding might reflect the fact that the main pathology during the early phase of CIDP is segmental demyelination, unless secondary axonal degeneration occurs (Boukhris et al., 2004; Harbo et al., 2008; Kuwabara et al., 2006; Said, 2006). In the early stages of CIDP, muscle structures tend to be preserved due to the limited denervation.

Reinnervation is another possible mechanism that might be preventing structural changes in muscles. However, the mechanism by which reinnervation affects MUS findings remains controversial. Specifically, Arts et al. revealed that some patients with amyotrophic lateral sclerosis exhibited increased MT and decreased EI in 6‐month sequential studies (Arts, Overeem, Pillen, Schelhaas, & Zwarts,^2011^). Zaidman et al. reported that while MT increased over time after nerve injury, EI remained elevated regardless of improvement in function in sequential studies of patients with newborn brachial plexus palsy (Zaidman, Holland, Noetzel, Park, & Pestronk, 2013). They speculated that the changes in MUS findings were associated with reinnervation to a certain extent; however, detailed mechanisms were not clarified. The preserved EI and MT values detected in this study may thus be attributed to reinnervation.

In recurrent cases with normal MUS findings, immune therapies such as IVIg, CS, and IS may have contributed to the prevention of secondary axonal degeneration and the promotion of reinnervation. Further studies are required to determine the mechanisms by which therapies may affect MUS findings.

### 4.3 Comparison of NCS and MUS findings

In this study, there was no significant difference in the dCMAP/LLN ratio between the six muscles with abnormal MUS findings suggesting axonal degeneration and the remaining muscles with normal MUS findings. This reflects the challenge in distinguishing secondary axonal degeneration from segmental demyelination in patients with CIDP by measuring dCMAP amplitudes in conventional NCSs as both pathologies could result in decreased dCMAP amplitudes (Harbo et al., 2008; Kuwabara et al., 2006, 2002; Tankisi et al., 2005, 2007). Based on this perspective, MUS should be considered as a complementary tool for NCSs in patients with CIDP as it can detect axonal degeneration by assessing muscle structural changes due to denervation.

We found fair correlations of dCMAP amplitude with both EI and MT in the CIDP group. Specifically, the dCMAP amplitude was negatively correlated with EI and positively correlated with MT (Figure 4). These findings were compatible with previous reports. Shahrizaila et al. reported that both cross‐sectional area and EI of the FDI were correlated with CMAP amplitude in patients with Charcot–Marie–Tooth neuropathy (Shahrizaila et al., 2017). Seok et al. reported that extensor digitorum brevis thickness was closely associated with fibular nerve CMAP amplitude (Seok, Walker, & Kwak, 2016). Our findings also demonstrated that quantitative MUS was a potentially complementary tool for NCS.

### 4.4 Limitations of the study

Our study has several limitations. First, the number of enrolled subjects was small. Evidently, future studies with larger sample sizes will be necessary to increase the statistical power. However, this study is the largest study examining MUS in patients with CIDP. Second, several variables, such as handedness and body mass index, which can affect MUS findings and in turn the study findings were not considered in our analyses (Arts, Pillen, Schelhaas, Overeem, & Zwarts, 2010; Simon et al., 2015). Third, correlations of MUS findings with needle EMG findings should be further studied to determine more accurate cut‐off values for accurate diagnosis of denervation. Fourth, only intrarater reliability was determined for quantitative MUS assessment and interrater reliability should be tested in a future study for its potential implementation in daily practice.

## 5 CONCLUSION

Quantitative MUS is a promising tool for detecting secondary axonal degeneration by determination of denervation in patients with CIDP. This approach can potentially be utilized to predict prognosis and responsiveness to treatment in patients with CIDP. Further studies with large populations and long‐period monitoring using objective clinical scales, such as the INCAT disability scale, are required to clarify the impact of MUS findings on the clinical course of patients with CIDP.

## CONFLICTS OF INTEREST

None declared.

## References

[brb3812-bib-0001] Arts, I. M. , Overeem, S. , Pillen, S. , Schelhaas, H. J. , & Zwarts, M. J. (2011). Muscle changes in amyotrophic lateral sclerosis: A longitudinal ultrasonography study. Clinical Neurophysiology, 122, 623–628. 20810308 10.1016/j.clinph.2010.07.023

[brb3812-bib-0002] Arts, I. M. , Pillen, S. , Schelhaas, H. J. , Overeem, S. , & Zwarts, M. J. (2010). Normal values for quantitative muscle ultrasonography in adults. Muscle and Nerve, 41, 32–41. 19722256 10.1002/mus.21458

[brb3812-bib-0003] Bouchard, C. , Lacroix, C. , Planté, V. , Adams, D. , Chedru, F. , Guglielmi, J. M. , & Said, G. (1999). Clinicopathologic findings and prognosis of chronic inflammatory demyelinating polyneuropathy. Neurology, 52, 498–503. 10025777 10.1212/wnl.52.3.498

[brb3812-bib-0004] Boukhris, S. , Magy, L. , Kabore, R. , Mabrouk, T. , Li, Y. , Sindou, P. , … Vallat, J. M. (2004). Atypical electrophysiologic findings in chronic inflammatory demyelinating polyneuropathy (CIDP) – diagnosis confirmed by nerve biopsy. Neurophysiologie Clinique, 34, 71–79. 15130553 10.1016/j.neucli.2004.01.004

[brb3812-bib-0005] Cornblath, D. R. , Kuncl, R. W. , Mellits, E. D. , Quaskey, S. A. , Clawson, L. , Pestronk, A. , & Drachman, D. B. (1992). Nerve conduction studies in amyotrophic lateral sclerosis. Muscle and Nerve, 15, 1111–1115. 1406768 10.1002/mus.880151009

[brb3812-bib-0006] Gunreben, G. , & Bogdahn, U. (1991). Real‐time sonography of acute and chronic muscle denervation. Muscle and Nerve, 14, 654–664. 1922172 10.1002/mus.880140709

[brb3812-bib-0007] Hahn, A. F. , Bolton, C. F. , Zochodne, D. , & Feasby, T. E. (1996). Intravenous immunoglobulin treatment in chronic inflammatory demyelinating polyneuropathy. A double‐blind, placebo‐controlled, cross‐over study. Brain, 119, 1067–1077. 8813271 10.1093/brain/119.4.1067

[brb3812-bib-0008] Harbo, T. , Andersen, H. , & Jakobsen, J. (2008). Length‐dependent weakness and electrophysiological signs of secondary axonal loss in chronic inflammatory demyelinating polyradiculoneuropathy. Muscle Nerve, 38, 1036–1045. 18642356 10.1002/mus.21000

[brb3812-bib-0009] Heckmatt, J. Z. , Leeman, S. , & Dubowitz, V. (1982). Ultrasound imaging in the diagnosis of muscle disease. Journal of Pediatrics, 101, 656–660. 7131136 10.1016/s0022-3476(82)80286-2

[brb3812-bib-0010] Iijima, M. , Yamamoto, M. , Hirayama, M. , Tanaka, F. , Katsuno, M. , Mori, K. , … Sobue, G. (2005). Clinical and electrophysiologic correlates of IVIg responsiveness in CIDP. Neurology, 64, 1471–1475. 15851750 10.1212/01.WNL.0000158680.89323.F8

[brb3812-bib-0011] Joint Task Force of the EFNS and the PNS . (2010). European federation of neurological societies/peripheral nerve society guideline on management of multifocal motor neuropathy: Report of a joint task force of the European federation of neurological societies and the peripheral nerve society–first revision. Journal of the Peripheral Nervous System, 15, 295–301. 21199100 10.1111/j.1529-8027.2010.00290.x

[brb3812-bib-0012] Kim, J. S. , Seok, H. Y. , & Kim, B. J. (2016). The significance of muscle echo intensity on ultrasound for focal neuropathy: The median‐ to ulnar‐innervated muscle echo intensity ratio in carpal tunnel syndrome. Clinical Neurophysiology, 127, 880–885. 25998202 10.1016/j.clinph.2015.04.055

[brb3812-bib-0013] Kuwabara, S. , Misawa, S. , Mori, M. , Tamura, N. , Kubota, M. , & Hattori, T. (2006). Long term prognosis of chronic inflammatory demyelinating polyneuropathy: A five year follow up of 38 cases. Journal of Neurology, Neurosurgery and Psychiatry, 77, 66–70. 10.1136/jnnp.2005.065441 PMC2117396 16361595

[brb3812-bib-0014] Kuwabara, S. , Ogawara, K. , Misawa, S. , Mori, M. , & Hattori, T. (2002). Distribution patterns of demyelination correlate with clinical profiles in chronic inflammatory demyelinating polyneuropathy. Journal of Neurology, Neurosurgery and Psychiatry, 72, 37–42. 10.1136/jnnp.72.1.37 PMC1737682 11784822

[brb3812-bib-0015] Lee, H. , Jee, S. , Park, S. H. , Ahn, S. C. , Im, J. , & Sohn, M. K. (2016). Quantitative muscle ultrasonography in carpal tunnel syndrome. Annals of Rehabilitation Medicine, 40, 1048–1056. 28119835 10.5535/arm.2016.40.6.1048 PMC5256332

[brb3812-bib-0016] Logigian, E. L. , Kelly, J. J. , & Adelman, L. S. (1994). Nerve conduction and biopsy correlation in over 100 consecutive patients with suspected polyneuropathy. Muscle and Nerve, 17, 1010–1020. 8065388 10.1002/mus.880170908

[brb3812-bib-0017] Pillen, S. , Arts, I. M. , & Zwarts, M. J. (2008). Muscle ultrasound in neuromuscular disorders. Muscle and Nerve, 37, 679–693. 18506712 10.1002/mus.21015

[brb3812-bib-0018] Pillen, S. , Verrips, A. , van Alfen, N. , Arts, I. M. , Sie, L. T. , & Zwarts, M. J. (2007). Quantitative skeletal muscle ultrasound: Diagnostic value in childhood neuromuscular disease. Neuromuscular Disorders, 17, 509–516. 17537635 10.1016/j.nmd.2007.03.008

[brb3812-bib-0019] Rubin, D. I. (2012). Technical issues and potential complications of nerve conduction studies and needle electromyography. Neurologic Clinics, 30, 685710. 10.1016/j.ncl.2011.12.008 22361380

[brb3812-bib-0020] Said, G. (2006). Chronic inflammatory demyelinating polyneuropathy. Neuromuscular Disorders, 16, 293–303. 16631367 10.1016/j.nmd.2006.02.008

[brb3812-bib-0021] Seok, J. I. , Walker, F. O. , & Kwak, S. G. (2016). Evaluation of extensor digitorum brevis thickness in healthy subjects: A comparative analysis of nerve conduction studies and ultrasound scans. Clinical Neurophysiology, 127, 1664–1668. 26315368 10.1016/j.clinph.2015.07.025

[brb3812-bib-0022] Sghirlanzoni, A. , Solari, A. , Ciano, C. , Mariotti, C. , Fallica, E. , & Pareyson, D. (2000). Chronic inflammatory demyelinating polyradiculoneuropathy: Long‐term course and treatment of 60 patients. Neurological Sciences, 21, 31–37. 10938200 10.1007/s100720070116

[brb3812-bib-0023] Shahrizaila, N. , Noto, Y. , Simon, N. G. , Huynh, W. , Shibuya, K. , Matamala, J. M. , … Kiernan, M. C. (2017). Quantitative muscle ultrasound as a biomarker in Charcot‐Marie‐tooth neuropathy. Clinical Neurophysiology, 128, 227–232. 27940147 10.1016/j.clinph.2016.11.010

[brb3812-bib-0024] Simon, N. G. , Ralph, J. W. , Lomen‐Hoerth, C. , Poncelet, A. N. , Vucic, S. , & Kiernan, M. C. (2015). Quantitative ultrasound of denervated hand muscles. Muscle and Nerve, 52, 221–230. 25388871 10.1002/mus.24519

[brb3812-bib-0025] Tankisi, H. , Pugdahl, K. , Fuglsang‐Frederiksen, A. , Johnsen, B. , Carvalho, M. D. , Fawcett, P. R. , … Esteem Project (2005). Pathophysiology inferred from electrodiagnostic nerve tests and classification of polyneuropathies. Suggested guidelines. Clinical Neurophysiology, 116, 1571–1580. 15907395 10.1016/j.clinph.2005.04.003

[brb3812-bib-0026] Tankisi, H. , Pugdahl, K. , Johnsen, B. , & Fuglsang‐Frederiksen, A. (2007). Correlations of nerve conduction measures in axonal and demyelinating polyneuropathies. Clinical Neurophysiology, 118, 2383–2392. 17900975 10.1016/j.clinph.2007.07.027

[brb3812-bib-0027] Van Asseldonk, J. T. , Van den Berg, L. H. , Kalmijn, S. , Wokke, J. H. , & Franssen, H. (2005). Criteria for demyelination based on the maximum slowing due to axonal degeneration, determined after warming in water at 37 degrees C: Diagnostic yield in chronic inflammatory demyelinating polyneuropathy. Brain, 128, 880–891. 15689367 10.1093/brain/awh375

[brb3812-bib-0028] Walker, F. O. , Cartwright, M. S. , Wiesler, E. R. , & Caress, J. (2004). Ultrasound of nerve and muscle. Clinical Neurophysiology, 115, 495–507. 15036045 10.1016/j.clinph.2003.10.022

[brb3812-bib-0029] Zaidman, C. M. , Holland, M. R. , Noetzel, M. J. , Park, T. S. , & Pestronk, A. (2013). Newborn brachial plexus palsy: Evaluation of severity using quantitative ultrasound of muscle. Muscle and Nerve, 47, 246–254. 23169008 10.1002/mus.23518

